# Supplementary material for: SmartPill™ Administration to Assess Gastrointestinal Function after Spinal Cord Injury in a Porcine Model—A Preliminary Study
Source: Biomedicines. 2023 Jun 7;11(6):1660. doi: 10.3390/biomedicines11061660 (PMC10295578; doi:10.3390/biomedicines11061660)
Supplement: Supplementary file 1 [file biomedicines-11-01660-s001.zip › biomedicines-2426060-supplementary.pdf]

Supplementary materials

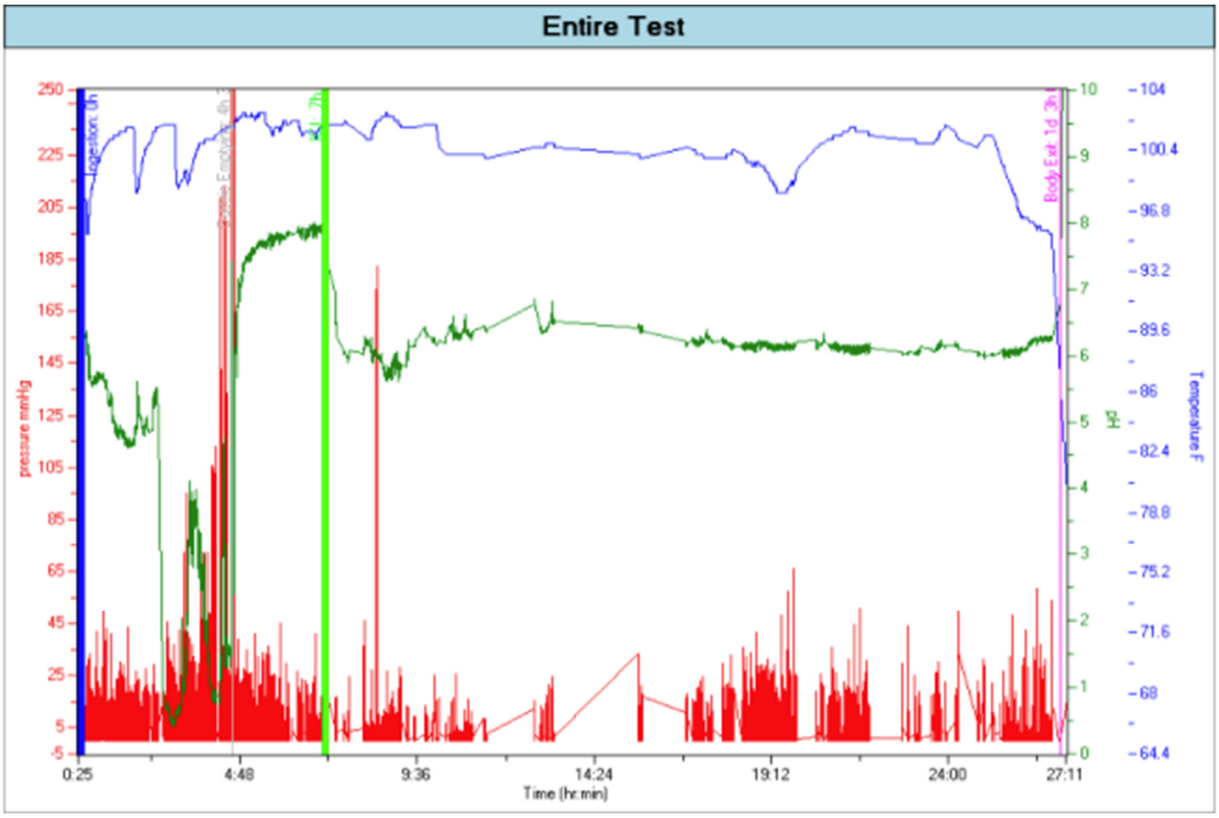

Supplemental Figure S1: SmartPill® pre-injury graph generated by MotiliGI® software for pig 1. The vertical green line indicates the ileocecal junction, the vertical red line indicates gastric emptying time (GET), and purple line represents body exit time. The precipitous fall in pH indicates that the WMC has been ingested and is located in the stomach. A permanent rise to greater than 4 pH to approximately 8 - 9 pH with a congruent increase in pressures indicate GET and passage through the antrum and pyloric sphincter and into the small intestines. Subsequent decrease in pH to approximately 6 - 8 with concomitant increases in pressure, indicates passage through the ileocecal valve and into the colon.

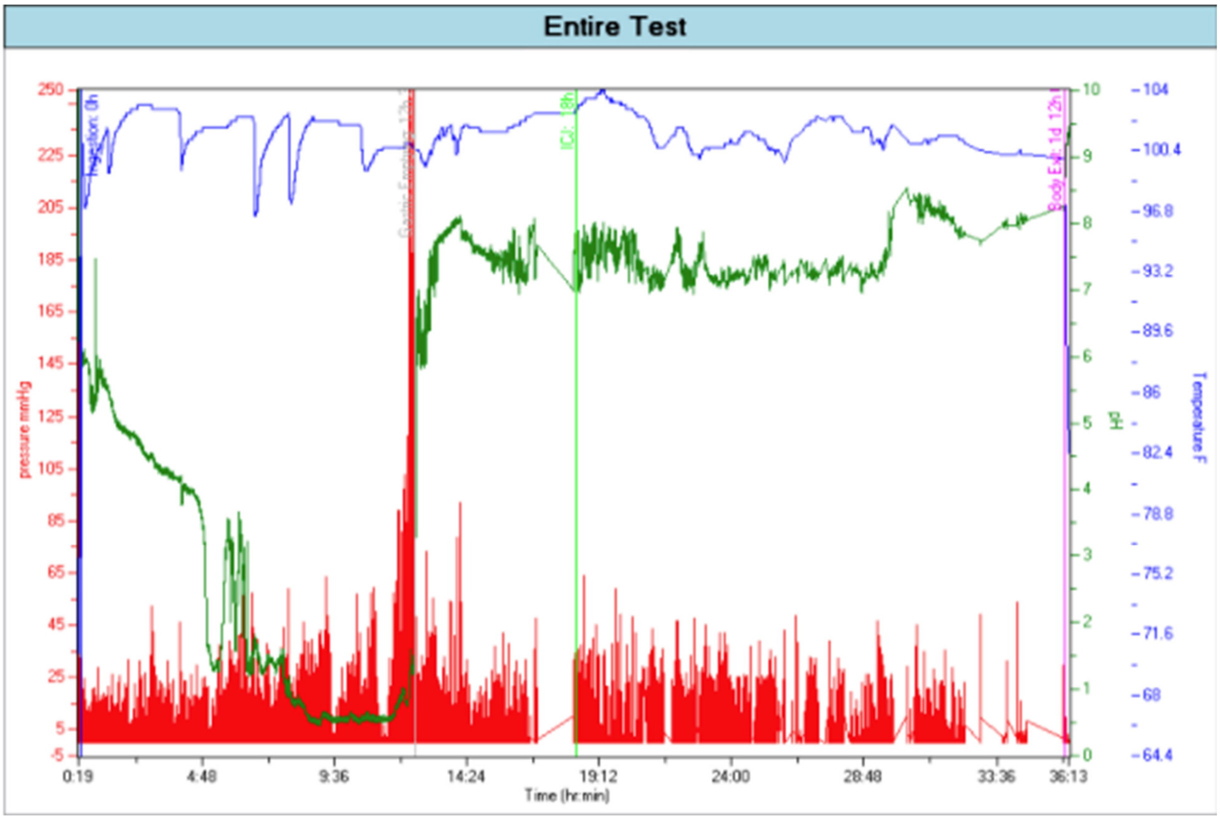

Supplemental Figure S2: SmartPill® 2 week post-injury graph generated by MotiliGI® software for pig 1. The vertical green line indicates the ileocecal junction, the vertical red line indicates gastric emptying time (GET), and purple line represents body exit time. The precipitous fall in pH indicates that the WMC has been ingested and is located in the stomach. A permanent rise to greater than 4 pH to approximately 8 - 9 pH with a congruent increase in pressures indicate GET and passage through the antrum and pyloric sphincter and into the small intestines. Subsequent decrease in pH to approximately 6 - 8 with concomitant increases in pressure, indicates passage through the ileocecal valve and into the colon.

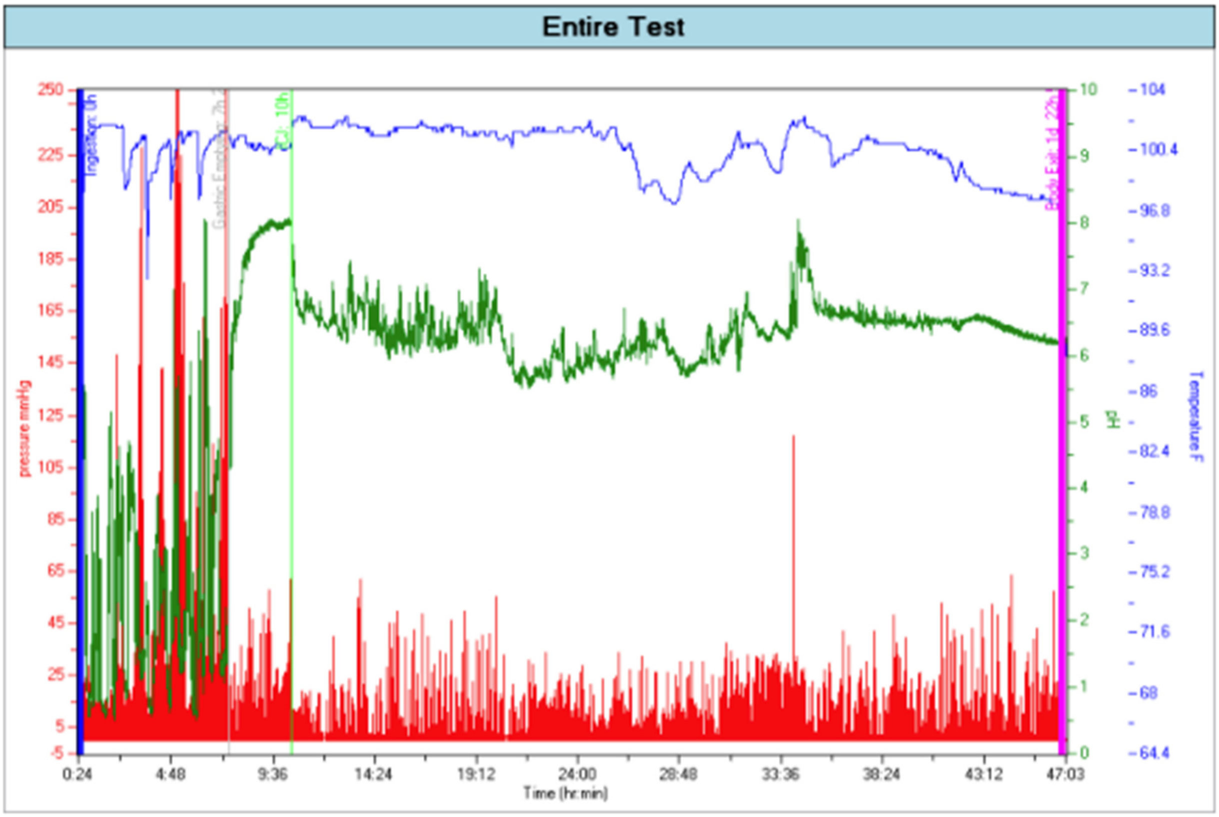

Supplemental Figure S3: SmartPill® 6-week post-injury graph generated by MotiliGI® software for pig 1. The vertical green line indicates the ileocecal junction, the vertical red line indicates gastric emptying time (GET), and purple line represents body exit time. The precipitous fall in pH indicates that the WMC has been ingested and is located in the stomach. A permanent rise to greater than 4 pH to approximately 8 - 9 pH with a congruent increase in pressures indicate GET and passage through the antrum and pyloric sphincter and into the small intestines. Subsequent decrease in pH to approximately 6 - 8 with concomitant increases in pressure, indicates passage through the ileocecal valve and into the colon.

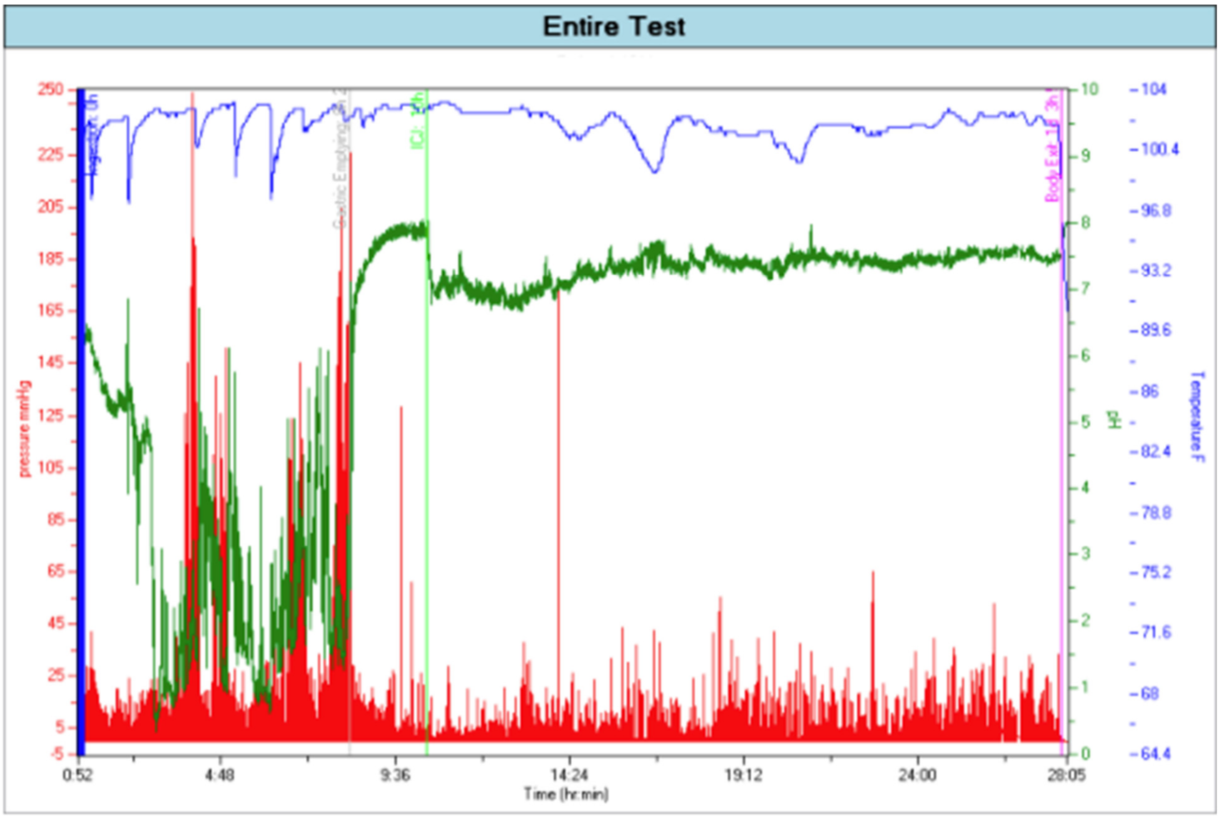

Supplemental Figure S4: SmartPill® pre-injury graph generated by MotiliGI® software for pig 2. The vertical green line indicates the ileocecal junction, the vertical red line indicates gastric emptying time (GET), and purple line represents body exit time. The precipitous fall in pH indicates that the WMC has been ingested and is located in the stomach. A permanent rise to greater than 4 pH to approximately 8 - 9 pH with a congruent increase in pressures indicate GET and passage through the antrum and pyloric sphincter and into the small intestines. Subsequent decrease in pH to approximately 6 - 8 with concomitant increases in pressure, indicates passage through the ileocecal valve and into the colon.

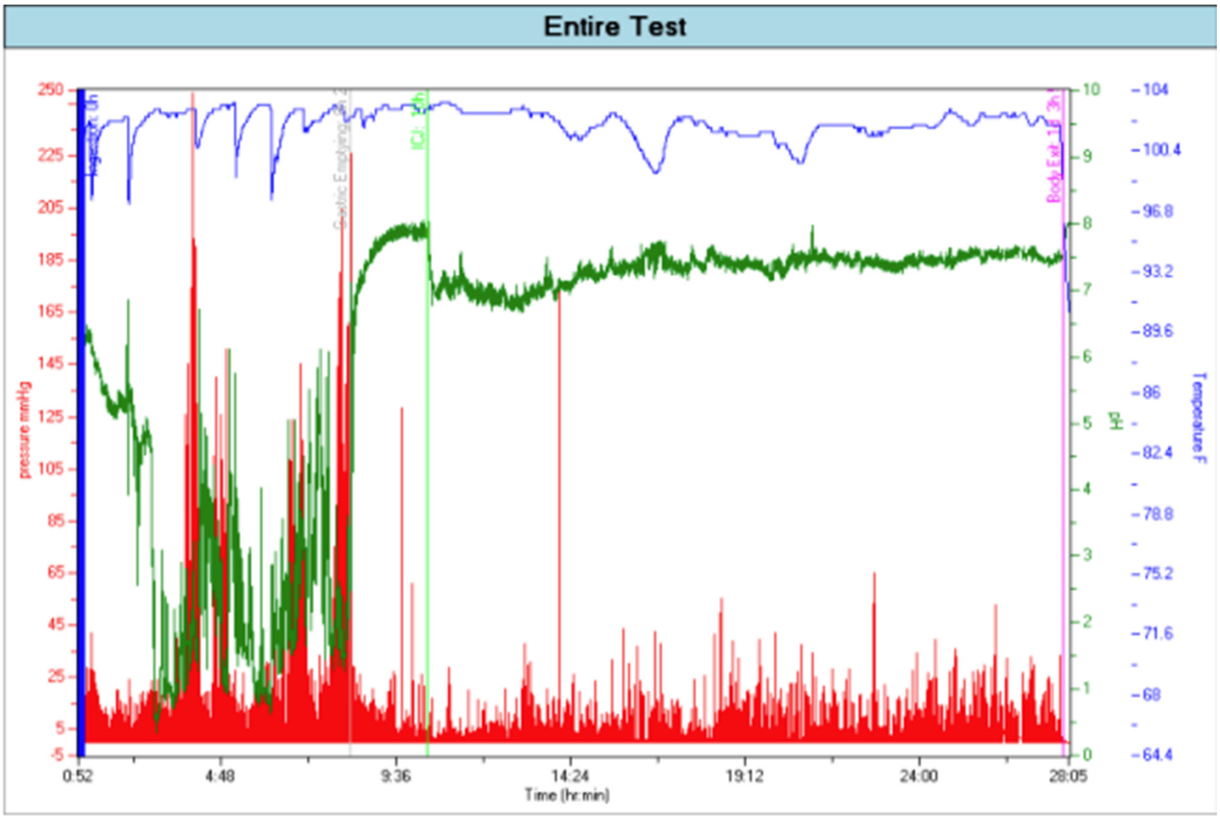

Supplemental Figure S5: SmartPill® 2-week post-injury graph generated by MotiliGI® software for pig 2. The vertical green line indicates the ileocecal junction, the vertical red line indicates gastric emptying time (GET), and purple line represents body exit time. The precipitous fall in pH indicates that the WMC has been ingested and is located in the stomach. A permanent rise to greater than 4 pH to approximately 8 - 9 pH with a congruent increase in pressures indicate GET and passage through the antrum and pyloric sphincter and into the small intestines. Subsequent decrease in pH to approximately 6 - 8 with concomitant increases in pressure, indicates passage through the ileocecal valve and into the colon.

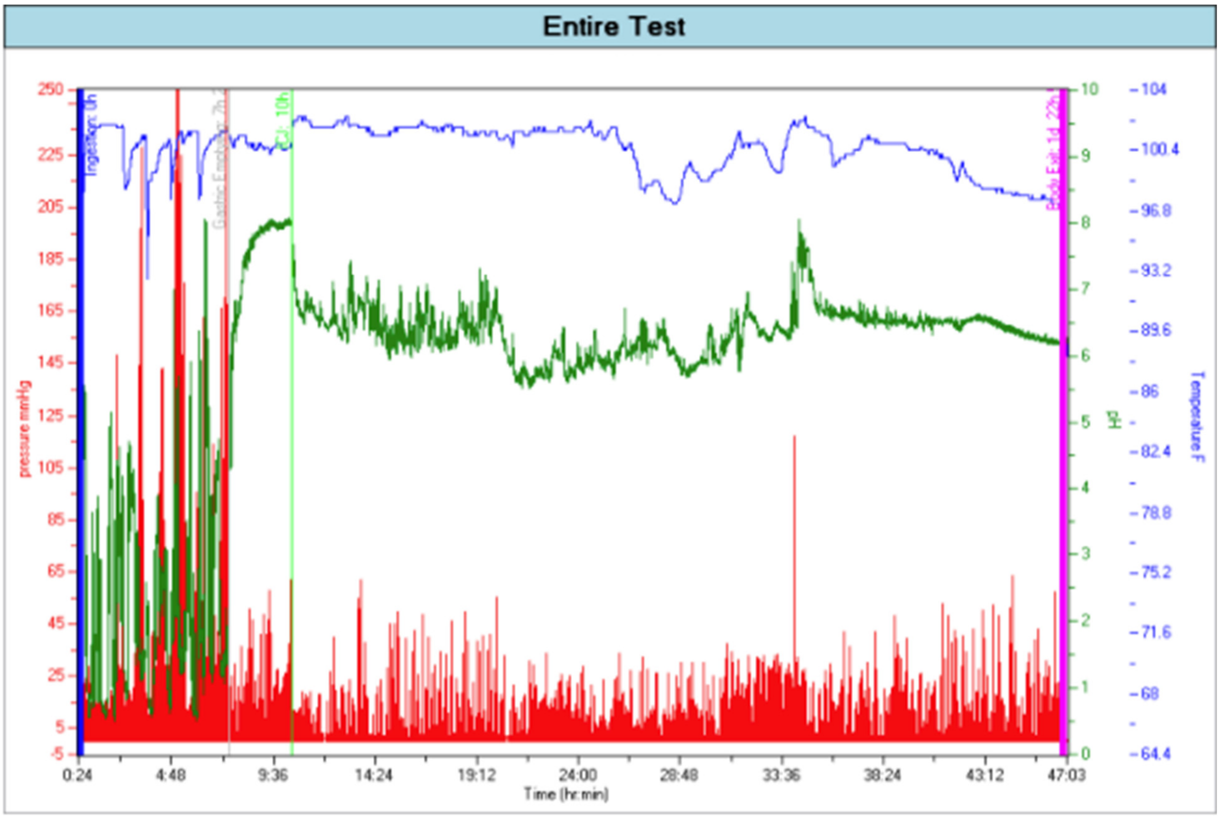

Supplemental Figure S6: SmartPill® 6-week post-injury graph generated by MotiliGI® software for pig 2. The vertical green line indicates the ileocecal junction, the vertical red line indicates gastric emptying time (GET), and purple line represents body exit time. The precipitous fall in pH indicates that the WMC has been ingested and is located in the stomach. A permanent rise to greater than 4 pH to approximately 8 - 9 pH with a congruent increase in pressures indicate GET and passage through the antrum and pyloric sphincter and into the small intestines. Subsequent decrease in pH to approximately 6 - 8 with concomitant increases in pressure, indicates passage through the ileocecal valve and into the colon.

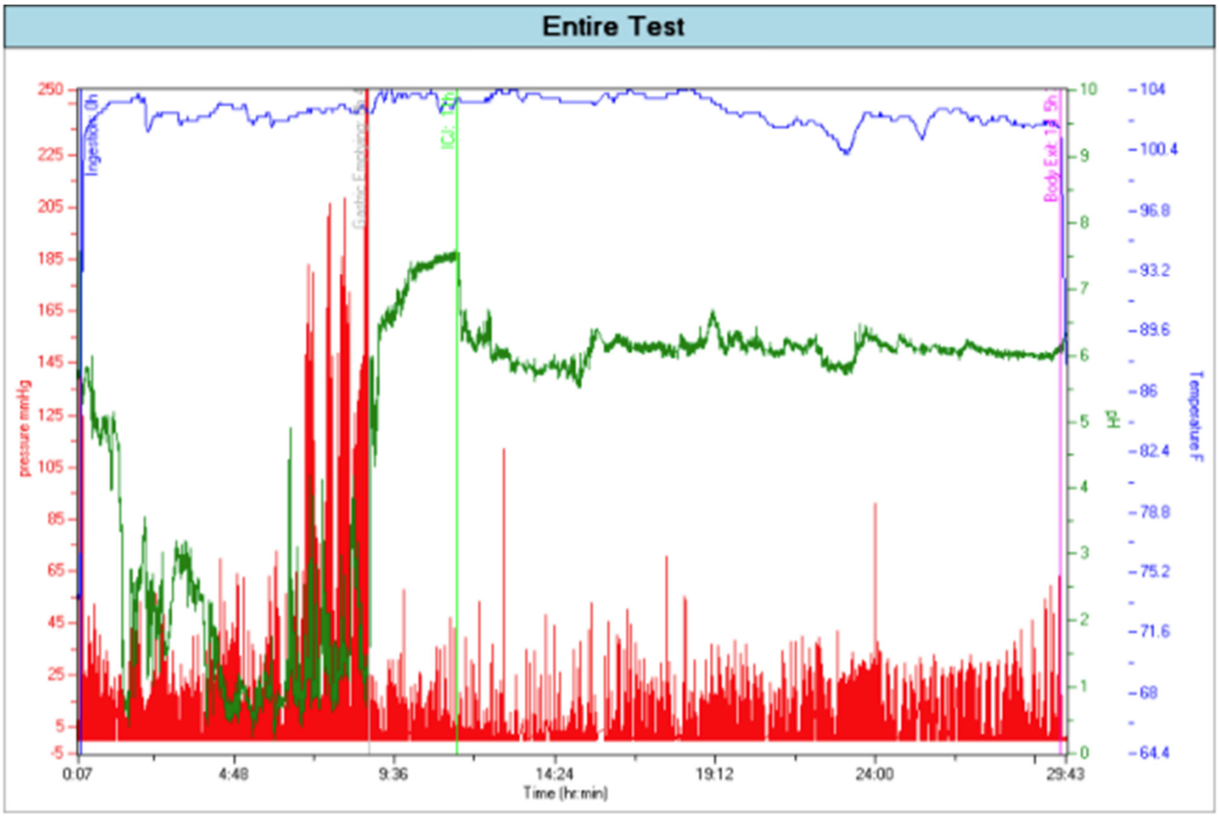

Supplemental Figure S7: SmartPill® 2-week post-injury graph generated by MotiliGI® software for pig 3. The vertical green line indicates the ileocecal junction, the vertical red line indicates gastric emptying time (GET), and purple line represents body exit time. The precipitous fall in pH indicates that the WMC has been ingested and is located in the stomach. A permanent rise to greater than 4 pH to approximately 8 - 9 pH with a congruent increase in pressures indicate GET and passage through the antrum and pyloric sphincter and into the small intestines. Subsequent decrease in pH to approximately 6 - 8 with concomitant increases in pressure, indicates passage through the ileocecal valve and into the colon.

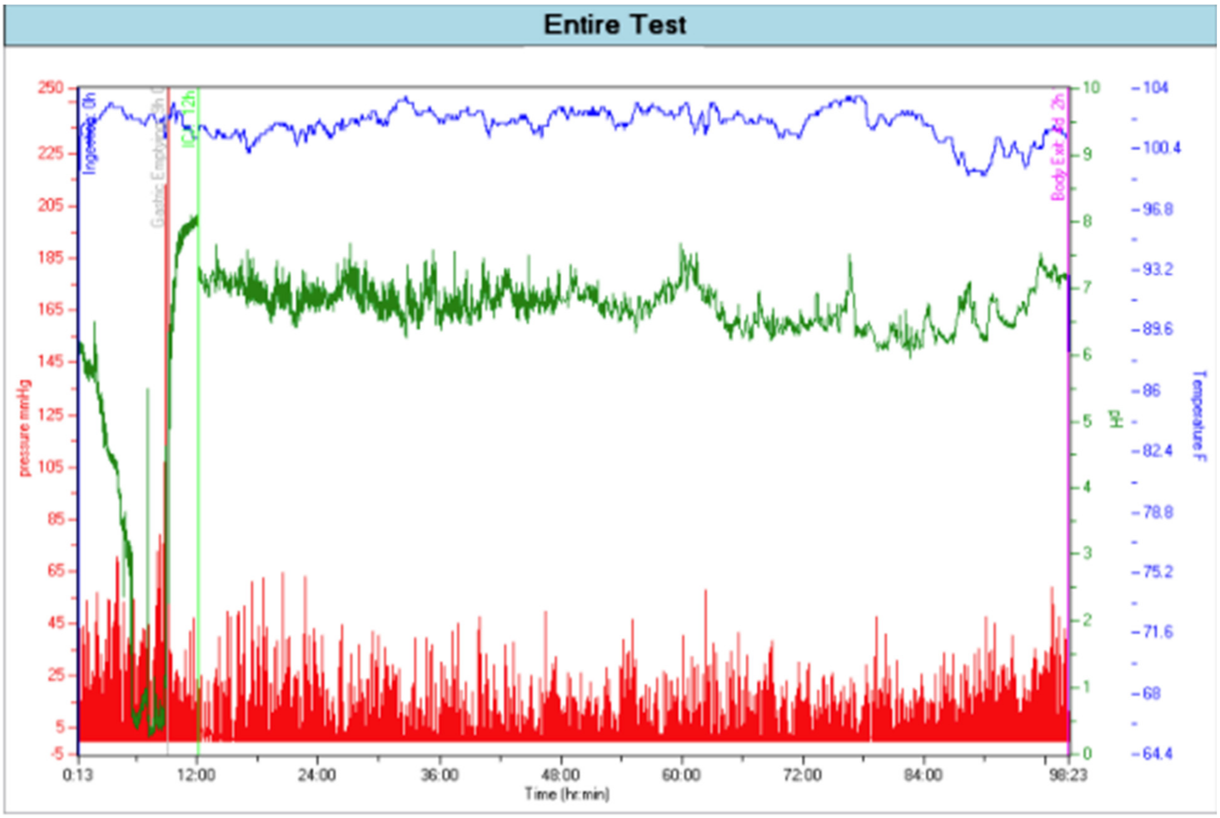

Supplemental Figure S8: SmartPill® 6-week post-injury graph generated by MotiliGI® software for pig 3. The vertical green line indicates the ileocecal junction, the vertical red line indicates gastric emptying time (GET), and purple line represents body exit time. The precipitous fall in pH indicates that the WMC has been ingested and is located in the stomach. A permanent rise to greater than 4 pH to approximately 8 - 9 pH with a congruent increase in pressures indicate GET and passage through the antrum and pyloric sphincter and into the small intestines. Subsequent decrease in pH to approximately 6 - 8 with concomitant increases in pressure, indicates passage through the ileocecal valve and into the colon.

Supplemental Table S1: Stomach [1]min and [2]med pH recordings stratified by time quartiles of stomach and anatomical regions similarly to other GI sections for each animal at each timepoint when the 3WMC was administered. We observed no appreciable trends in pH over the six-week study. [1]Minimum, [2]Median, [3]Wireless Motility Capsule.

Stomach pH											
Pig	Time	Antrum		Section 1		Section 2		Section 3		Section 4	
		Min	Med	Min	Med	Min	Med	Min	Med	Min	Med
1	Pre-injury	0.73	1.5	4.75	5.58	4.61	4.86	0.43	0.71	0.73	1.52
	2 weeks	0.52	0.8	4.24	4.93	1.26	3.26	0.46	0.95	0.48	0.58
	6 weeks	1.07	1.76	0.5	1.6	0.56	1.98	0.62	2.26	0.5	1.58
2	Pre-injury	1.25	2.31	2.56	5.32	0.35	1.69	0.61	1.71	1.25	2.45
	2 weeks	3.73	5.41	0.31	2.61	0.8	3.48	1.33	3.46	1.78	3.94
	6 weeks	1.2	2.18	0.69	4.52	0.46	1.9	0.74	1.61	1.08	2.23
3	Pre-injury	0.71	1.5	0.4	4.08	0.42	2.18	0.2	0.84	0.41	1.39
	2 weeks	0.37	0.58	5.1	5.78	3.37	4.42	0.39	0.78	0.26	0.54
